# The value of radiographic features in predicting postoperative facial nerve function in vestibular schwannoma patients: A retrospective study and nomogram analysis

**DOI:** 10.1111/cns.14526

**Published:** 2023-11-21

**Authors:** Xudong Shi, Yuyang Liu, Zehan Zhang, Bingyan Tao, Ding Zhang, Qingyu Jiang, Guilin Chen, Hengchao Ma, Yaping Feng, Jiaxin Xie, Xuan Zheng, Jun Zhang

**Affiliations:** ^1^ Department of Neurosurgery, The First Medical Centre Chinese PLA General Hospital Beijing China; ^2^ Medical School of Chinese PLA Beijing China; ^3^ Department of Neurosurgery 920th Hospital of Joint Logistics Support Force Kunming China; ^4^ Department of Neurosurgery the Air Force Hospital of Southern Theater Command Guangzhou China; ^5^ Department of Neurosurgery 961th Hospital of Joint Logistics Support Force Qiqihar China

**Keywords:** facial paralysis, nomogram, predict, vestibular schwannoma

## Abstract

**Objective:**

The purpose of this study was to identify significant prognostic factors associated with facial paralysis after vestibular schwannoma (VS) surgery and develop a novel nomogram for predicting facial nerve (FN) outcomes.

**Methods:**

Retrospective data were retrieved from 355 patients who underwent microsurgery via the retrosigmoid approach for VS between December 2017 and December 2022. Univariate and multivariate logistic regression analysis were used to construct a radiographic features‐based nomogram to predict the risk of facial paralysis after surgery.

**Results:**

Following a thorough screening process, a total of 185 participants were included. The univariate and multivariate logistic regression analysis revealed that tumor size (*p* = 0.005), fundal fluid cap (FFC) sign (*p* = 0.014), cerebrospinal fluid cleft (CSFC) sign (*p* < 0.001), and expansion of affected side of internal auditory canal (IAC) (*p* = 0.033) were independent factors. A nomogram model was constructed based on these indicators. When applied to the validation cohort, the nomogram demonstrated good discrimination and favorable calibration. Then we generated a web‐based calculator to facilitate clinical application.

**Conclusion:**

Tumor size, FFC and CSFC sign, and the expansion of the IAC, serve as good predictors of postoperative FN outcomes. Based on these factors, the nomogram model demonstrates good predictive performance.

## Graphical abstract

The radiographic features, including tumor size, fundal fluid cap (FFC) and cerebrospinal fluid cleft (CSFC) sign, and the expansion of the IAC, may serve as good predictors of postoperative facial nerve (FN) outcomes after vestibular schwannoma resection.

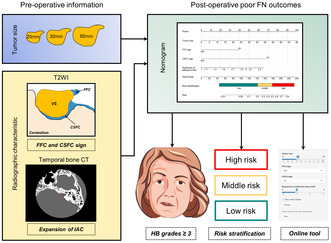

## 1 INTRODUCTION

Vestibular schwannoma is a benign nerve sheath tumor that originates from the Schwann cells of the vestibular cochlear nerve, accounting for 8% of all intracranial tumors and 85% of tumors in the cerebellopontine angle (CPA) region.^1^ The main treatment options for VS include observation, radiotherapy, and surgical treatment, with surgical resection currently being the primary approach. Facial paralysis is a common complication of surgical treatment due to the proximity of the facial nerve (FN) to the vestibular nerve. Postoperative facial paralysis after VS resection can lead to psychological issues, such as depression and social isolation, significantly impacting the quality of life.^2^

Predicting the postoperative FN function preoperatively would be helpful in guiding patients and determining treatment strategies. Currently, it is believed that several factors are associated with postoperative FN function in VS, including age, tumor size and cystic versus solid nature, etc., but the predictive value of some risk factors remains controversial.^3^ Various radiographic features have been shown to be associated with postoperative nerve function.^4^ For instance, several studies have reported that the presence of cerebrospinal fluid at the outermost end of the internal auditory canal (IAC) indicates preserved hearing after VS surgery, a radiological characteristic known as the fundal fluid cap (FFC).^5^, ^6^, ^7^ A recent study also demonstrated that FFC sign can predict postoperative FN function.^5^ Another radiological characteristic, the cerebrospinal fluid cleft (CSFC) sign, can assess the degree of adhesion between the VS and surrounding tissues.^8^ Sun et al. verified that the CSFC sign is an independent risk factor for FN prognosis and integrated it into a clinical prediction model.^3^ However, the applicability of this prediction model is questionable due to its subjective factors such as the learning curve.

In this study, we conducted a retrospective analysis of clinical and imaging data from our institution to develop a robust and clinically significant nomogram that utilizes preoperative clinical and imaging factors to predict the postoperative prognosis of FN function following VS surgical resection. Subsequently, we performed a comprehensive evaluation and validation of the predictive model.

## 2 METHODS

### 2.1 Patients

**FIGURE 1:**
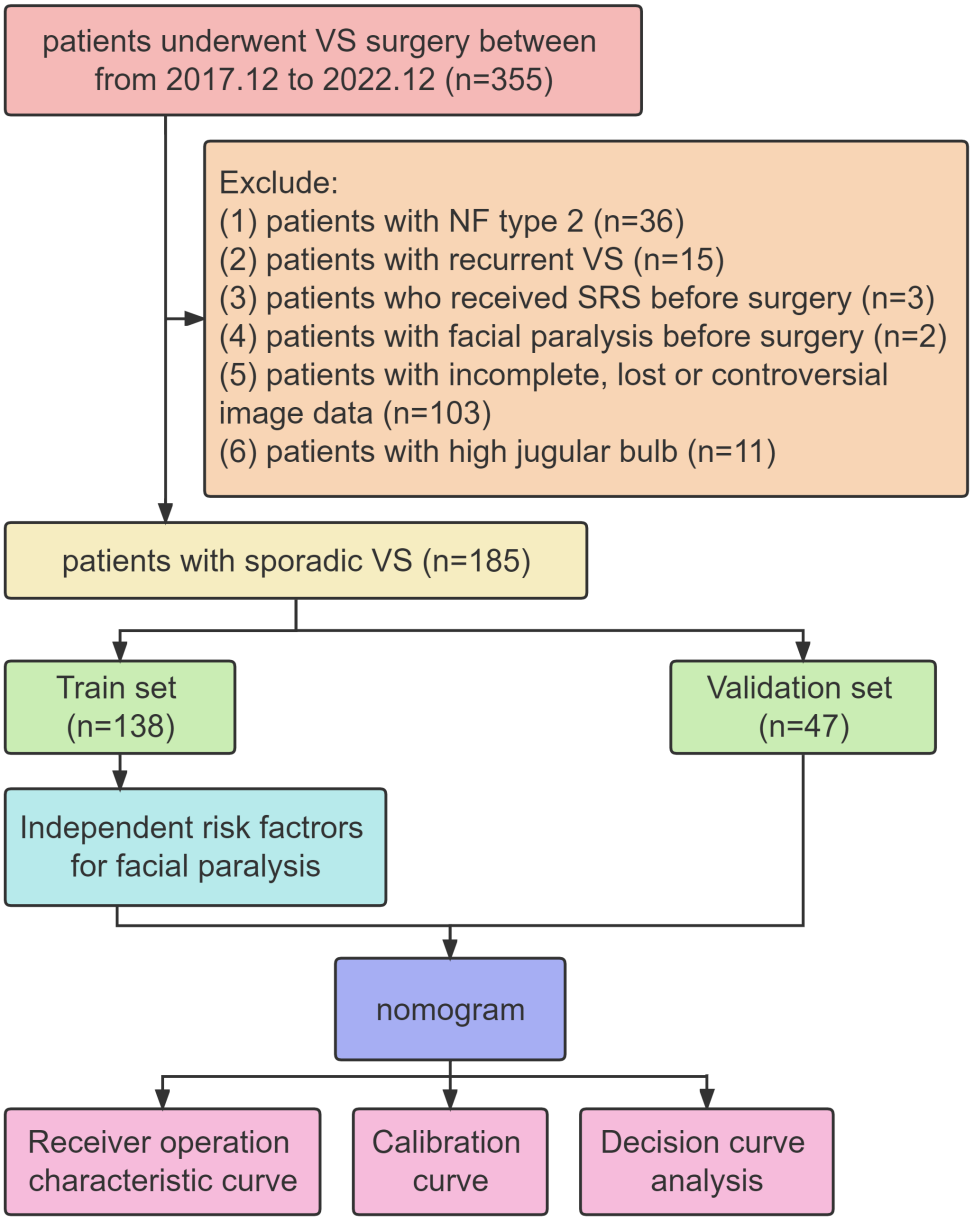
Flow chart of the study. NF, neurofibromatosis; SRS, stereotactic radiosurgery.

This study utilized a retrospective design and collected clinical data from patients who underwent surgical treatment for VS at the PLA General Hospital from December 2017 to December 2022. Continuous acquisition of relevant data was ensured. To be included, patients needed to meet the following criteria: (1) complete medical records that include clinical, imaging, and pathological details; (2) confirmation of VS diagnosis through pathology; and (3) receiving primary surgical treatment via the retrosigmoid approach (Figure 1). The study was approved by the ethics committee of our institution, and patient consent was waived due to the study design.

### 2.2 Demographic and clinical characteristics

Demographic and clinical information were obtained from the patients' medical records and radiology database. The clinical characteristics included age, gender, body mass index (BMI), tinnitus, trigeminal hypoesthesia, vertigo, imbalance, diabetes mellitus, high blood pressure (HBP), smoking history, frequent alcohol consumption, tumor side, preoperative hearing loss, and short‐term FN outcomes after surgery. According to the foundation of the American Academy of Otolaryngology‐Head and Neck Surgery, the serviceable hearing was defined as having a speech discrimination score (SDS) of ≥50% and a pure tone average (PTA) of ≤50 dB. The FN function was evaluated at discharge (days 5–8) using the House–Brackmann (HB) grading scale.^9^ HB grades ≥3 were categorized as poor FN outcomes.

### 2.3 Radiographic characteristic on MRI and CT

The radiographic characteristics were extracted from the PLA general hospital image database, subjectively investigated by two independent neurosurgeons blindly. These included FFC sign, CSFC sign, brainstem compression, tumor size, cystic features of tumors, tumor homogeneity, hydrocephalus, affected side of IAC, normal side of IAC, expansion of affected side of IAC, and diameter of the fourth ventricle.

**FIGURE 2:**
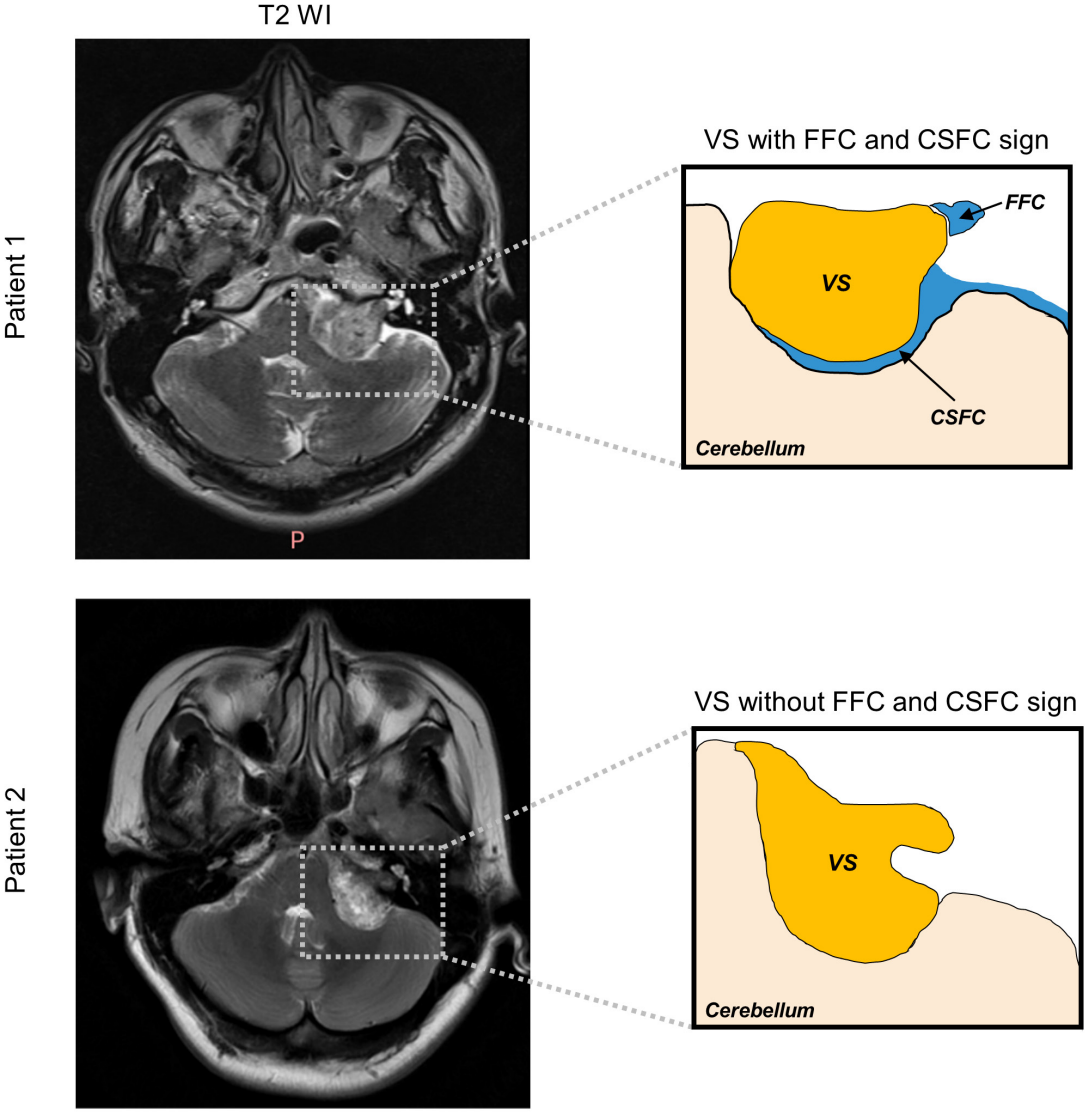
Patient 1 shows a VS with FFC and CSFC sign, and patient 2 shows a VS without FFC and CSFC sign.

Tumor size was assessed by measuring the largest diameter of the tumor in the CPA excluding the intracanalicular component of the lesion. Intratumoral cysts were defined as regions bounded by a contrast‐enhancing wall and contents hypointense on T1‐weighted gadolinium‐enhanced magnetic resonance imaging (Gd‐T1wMRI), while appearing hyperintense on T2‐weighted magnetic resonance imaging (T2wMRI). Along with a range of well‐established preoperative radiographic factors, several recently described factors are defined as follows. The FFC sign was determined as the presence of CSF in the most lateral end of a tumor in the IAC on T2wMRI (Figure 2).^5^ The CSFC sign was determined as the presence of a CSFC around 50% of the tumor on T2wMRI (Figure 2).^4^ Affected/normal side of IAC was determined as distance from the nasal crest to the dorsal crest of the IAC at the affected/normal side. Expansion of affected side of IAC was determined as the ratio of distance of the affected side to distance of the healthy side and those above were measured on temporal bone CT.^10^

### 2.4 Statistical analysis

In our study, we utilized version 22.0 of the SPSS software program (IBM Corporation, Armonk, NY, USA), to check the correlation matrix of all potential predictive factors and avoid multicollinearity. To identify potential risk factors, we conducted univariate and multivariate logistic regression analysis using R software, version 4.2.2 (http://www.r‐project.org/). A backward stepwise regression method, specifically the step AIC function from the MASS package (v7.3‐57), was used to select the optimal combination of variables from the univariate analysis for inclusion in the multivariate analysis model. Then we conducted the nomogram model (rsm package, v6.6–0) and evaluated the performance through receiver operating characteristic (ROC) analysis (pROC package, v1.18.0), calibration curves (rsm package, v 6.6‐0), and decision curve analysis (DCA, ggDCA pakeage, v1.2). A *p*‐value of less than 0.05 was considered statistically significant for all conducted analysis.

## 3 RESULTS

### 3.1 Demographic and clinical Variables

The cohort of 185 patients who underwent VS surgery and fulfilled the inclusion criteria were included in the analysis. All the demographic information and baseline characteristics of the included patients are presented in Table 1. The training set was composed of 138 patients, whereas the validation set encompassed 47 patients.

**TABLE 1:** Patient demographics and baseline characteristic.

Characteristic	Facial nerve outcome, *n* (%)			*p*‐value^a^
	Overall (*N* = 185)	Good (*N* = 145)	Poor (*N* = 40)	
Gender				
Female	102 (55%)	80 (55%)	22 (55%)	0.985
Male	83 (45%)	65 (45%)	18 (45%)	
Age				
Median (IQR)	50 (40, 56)	50 (40, 56)	46 (38, 54)	0.349
BMI				
Median (IQR)	24.0 (21.4, 26.4)	23.9 (21.3, 26.4)	24.4 (21.7, 25.9)	0.754
Tinnitus				
No	68 (37%)	51 (35%)	17 (43%)	0.395
Yes	117 (63%)	94 (65%)	23 (58%)	
Trigeminal hypoesthesia				
No	131 (71%)	108 (74%)	23 (58%)	0.036
Yes	54 (29%)	37 (26%)	17 (43%)	
Vertigo				
No	133 (72%)	104 (72%)	29 (73%)	0.923
Yes	52 (28%)	41 (28%)	11 (28%)	
Imbalance				
No	154 (83%)	122 (84%)	32 (80%)	0.535
Yes	31 (17%)	23 (16%)	8 (20%)	
Diabetes mellitus				
No	179 (97%)	139 (96%)	40 (100%)	0.343
Yes	6 (3.2%)	6 (4.1%)	0 (0%)	
HBP				
No	147 (79%)	115 (79%)	32 (80%)	0.924
Yes	38 (21%)	30 (21%)	8 (20%)	
Smoker				
No	162 (88%)	127 (88%)	35 (88%)	>0.999
Yes	23 (12%)	18 (12%)	5 (13%)	
Frequent alcohol consumption				
No	170 (92%)	132 (91%)	38 (95%)	0.530
Yes	15 (8.1%)	13 (9.0%)	2 (5.0%)	
Side				
Right	94 (51%)	76 (52%)	18 (45%)	0.406
Left	91 (49%)	69 (48%)	22 (55%)	
Serviceable hearing				
Yes	58 (31%)	45 (31%)	13 (33%)	0.860
No	127 (69%)	100 (69%)	27 (68%)	
FFC sign				
Yes	79 (43%)	73 (50%)	6 (15%)	
No	106 (57%)	72 (50%)	34 (85%)	
CSFC sign				
Yes	110 (59%)	104 (72%)	6 (15%)	<0.001
No	75 (41%)	41 (28%)	34 (85%)	
Brainstem compression				
No	67 (36%)	64 (44%)	3 (7.5%)	<0.001
Yes	118 (64%)	81 (56%)	37 (93%)	
Tumor size (mm)				
Median (IQR)	24 (15, 32)	21 (13, 27)	35 (30, 40)	<0.001
Cystic				
No	124 (67%)	106 (73%)	18 (45%)	<0.001
Yes	61 (33%)	39 (27%)	22 (55%)	
Tumor homogeneity				
No	85 (46%)	60 (41%)	25 (63%)	0.018
Yes	100 (54%)	85 (59%)	15 (38%)	
Hydrocephalus				
No	152 (82%)	128 (88%)	24 (60%)	<0.001
Yes	33 (18%)	17 (12%)	16 (40%)	
Affected side of IAC (mm)				
Median (IQR)	10.4 (9.1, 12.1)	10.3 (9.0, 12.1)	11.0 (9.3, 12.2)	0.217
Normal side of IAC (mm)				
Median (IQR)	7.5 (6.5, 8.6)	7.6 (6.5, 8.6)	7.4 (6.3, 8.3)	0.300
Expansion of affected side of IAC (mm)				
Median (IQR)	1.4 (1.2, 1.6)	1.4 (1.2, 1.6)	1.5 (1.3, 1.8)	0.029
Diameter of the fourth ventricle (mm)				
Median (IQR)	11.7 (9.1, 13.1)	11.8 (9.6, 13.4)	10.0 (5.9, 12.0)	<0.001

^a^ Pearson's chi‐squared test; Wilcoxon rank sum test; Fisher's exact test.

### 3.2 Univariate and multivariate logistic regression analysis of FN outcomes after VS surgery

In the train set, statistically significant differences were found in trigeminal hypoesthesia, FFC sign, CSFC sign, brainstem compression, tumor size, cystic feature, tumor homogeneity, hydrocephalus, expansion of affected side of IAC, and diameter of the fourth ventricle. Among them, tumor size (*p* = 0.005), FFC sign (*p* = 0.014), CSFC sign (*p* < 0.001), and expansion of affected side of IAC (*p* = 0.033) were identified as independent risk factors for poor FN outcomes by the multivariate regression analysis. (*p* < 0.05; Table 2).

**TABLE 2:** Univariate and multivariate logistic regression in the train set.

Characteristic	Univariate analysis			Multivariate analysis		
	OR	95% CI	*p*‐value	OR	95% CI	*p*‐value
Gender						
Female	—	—				
Male	1.15	0.47,2.80	0.754			
Age	1.00	0.97, 1.04	0.926			
BMI	1.00	0.88, 1.12	0.944			
Tinnitus						
No	—	—				
Yes	0.67	0.27, 1.69	0.389			
Trigeminal hypoesthesia						
No	—	—				
Yes	2.73	1.08, 6.81	0.031			
Vertigo						
No	—	—				
Yes	0.57	0.18, 1.55	0.300			
Imbalance						
No	—	—				
Yes	0.52	0.08, 1.99	0.402			
Diabetes mellitus						
No	—	—				
Yes	0.00		0.989			
HBP						
No	—	—				
Yes	0.89	0.24, 2.65	0.839			
Smoker						
No	—	—				
Yes	1.32	0.35, 4.10	0.651			
Frequent alcohol consumption						
No	—	—				
Yes	0.34	0.02, 1.83	0.307			
Tumor side						
Right	—	—				
Left	2.07	0.84, 5.47	0.123			
Serviceable hearing						
No	—	—				
Yes	1.15	0.45, 2.84	0.759			
FFC sign						
No	—	—		—	—	
Yes	0.24	0.07, 0.63	0.007	0.18	0.04, 0.66	0.014
CSFC sign						
No	—	—		—	—	
Yes	0.06	0.01, 0.18	<0.001	0.07	0.01, 0.26	<0.001
Brainstem compression						
No	—	—				
Yes	8.29	2.29, 53.31	0.006			
Tumor size	1.11	1.06, 1.16	<0.001	1.09	1.03, 1.17	0.005
Cystic						
No	—	—				
Yes	5.60	2.23, 15.09	<0.001			
Tumor homogeneity						
No	—	—				
Yes	0.33	0.12, 0.80	0.018			
Hydrocephalus						
No	—	—				
Yes	5.10	1.81, 14.24	0.002			
Expansion of affected side of IAC	4.77	1.15, 20.70	0.032	8.73	1.23, 69.48	0.033
Diameter of the fourth ventricle	0.84	0.73, 0.96	0.014			

*Note*: Number in dataframe = 138, Number in model = 138, AIC = 84.8, *C*‐statistic = 0.93, H&L = Chi‐sq (8) 4.27 (*p* = 0.832).

Abbreviations: CI, confidence interval; OR, odds ratio.

### 3.3 Nomogram of FN outcomes after VS surgery

**FIGURE 3:**
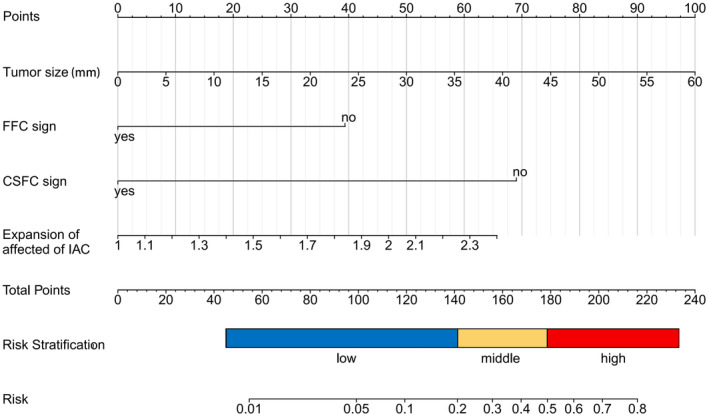
Nomogram for predicting the probability of poor FN outcome in patients with VSs.

The nomogram for predicting poor FN outcomes is shown in Figure 3. The development of this nomogram involved four distinct predictive factors, namely FFC sign, CSFC sign, tumor size, and expansion of the affected side of IAC. Each of these factors was assigned a specific weightage in terms of points, and the cumulative points corresponding to each patient were linked to a precise forecast of FN outcomes.

**FIGURE 4:**
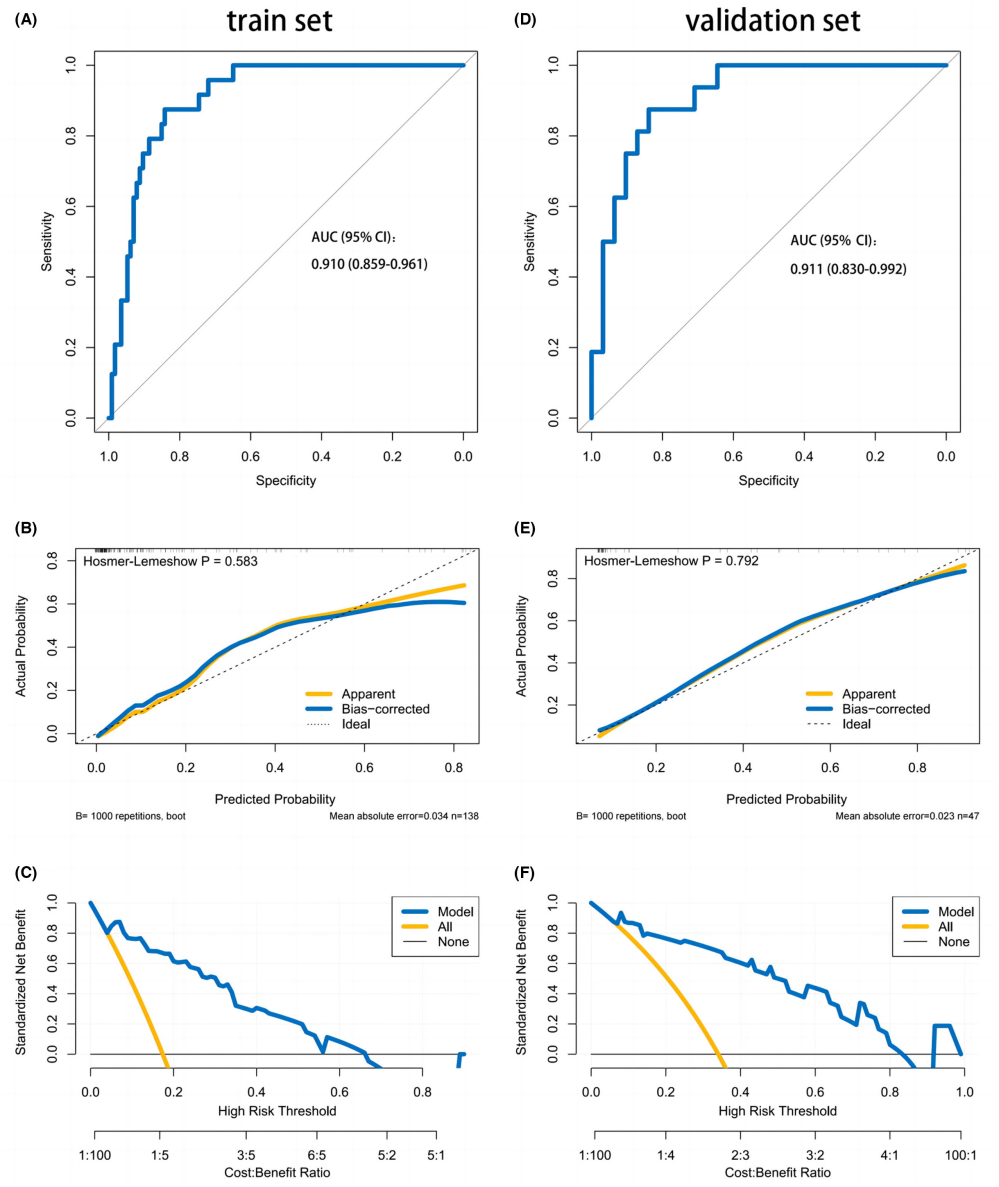
ROC curves of the nomogram for the training set (A) and validation set (D) (AUC, areas under the curve). Calibration curves of the nomogram prediction in the train set (B) and validation set (E) (*p* < 0.05 means significant). The decision curve analysis for the nomogram in the training set (C) and validation set (F).

The nomogram demonstrated favorable discrimination with an area under curve (AUC) of 0.910 (95% CI: 0.859–0.961) in the training set (Figure 4A). The calibration curve indicated a strong correlation between the model's predictions and the actual observations in the training set (Figure 4B). The Hosmer–Lemeshow test yielded a nonsignificant *p* = 0.583, indicating that the predicted and actual probability were highly consistent. The DCA demonstrated that using the nomogram to determine whether to pursue surgery was more beneficial compared to treating all patients or no patients in the training set when the threshold probabilities for clinicians or patients fell within the range of 5%–70% (Figure 4C). This study clearly establishes the value of our nomogram as a promising tool for guiding clinical decision‐making. The discrimination of the nomogram was confirmed to be satisfactory using the validation set, with an AUC of 0.911 (95% CI: 0.830–0.992; Figure 4D). Similarly, in the validation set, we observed good calibration, as indicated by a nonsignificant *p*‐value of 0.792 obtained from the Hosmer–Lemeshow test. (Figure 4E). The DCA indicated that in the validation set, the nomogram exhibited a higher net benefit when the threshold probabilities were in the range of 5%–84% (Figure 4F).

**FIGURE 5:**
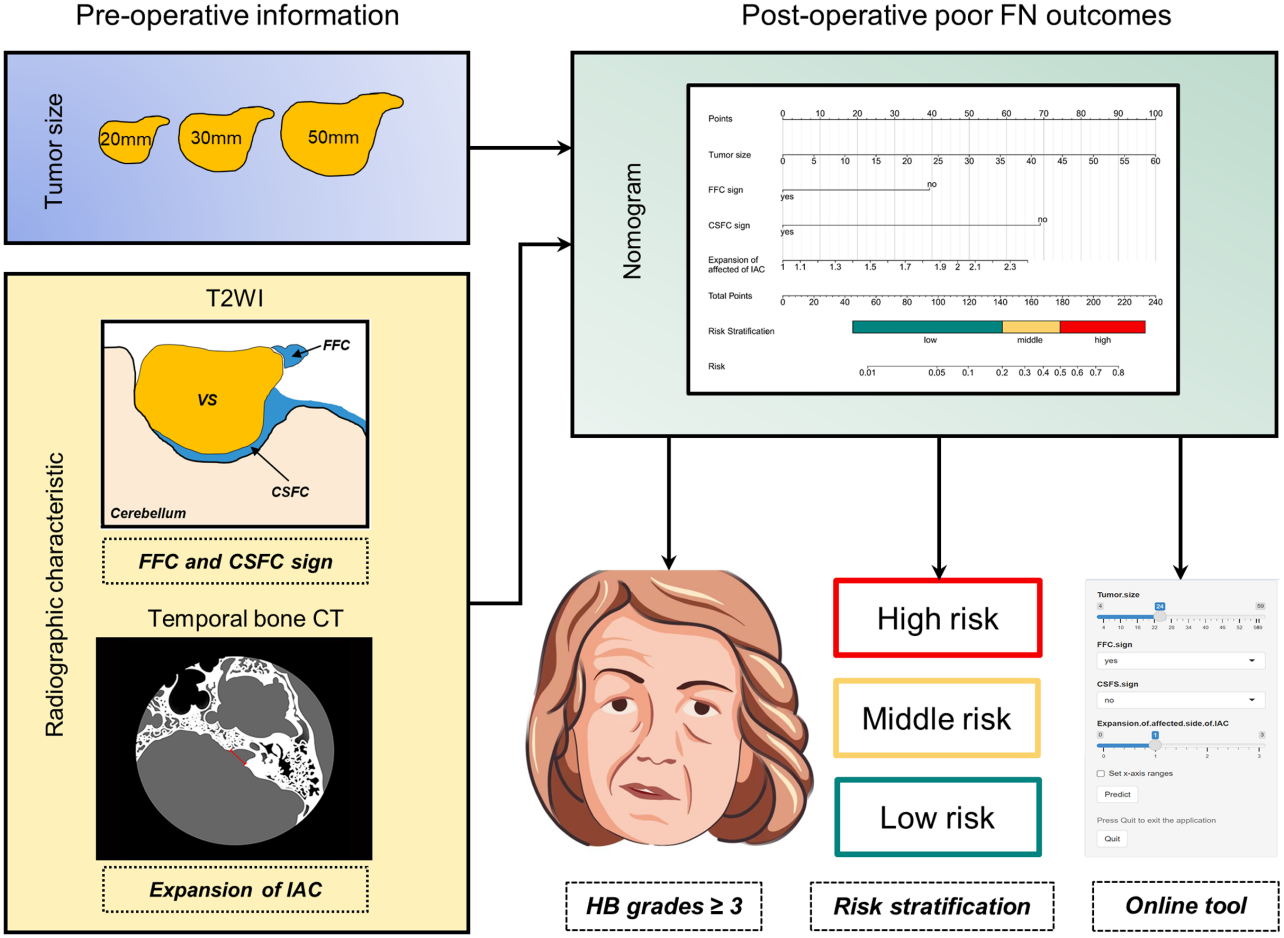
Summary of this analysis.

Overall analysis was briefly presented in Figure 5.

## 4 DISCUSSION

Accurately assessing the risk of facial paralysis is crucial for counseling patients considering microsurgical resection of VS and predicting the postoperative FN function preoperatively holds immense significance. In this study, approximately 21.6% of patients had poor FN outcomes (H‐B grade III‐VI) at discharge. We found that patients with VSs without FFC or CSFC signs, and with larger tumor volumes and greater IAC dilation, were more likely to FN dysfunction after surgery. Based on these imaging predictive factors, the columnar nomogram model was successfully constructed to predict the individualized risk of unfavorable FN outcomes after surgery in patients with VSs.

FN dysfunction following VS resection is related to multiple factors.^11^ It is universally acknowledged that tumor size is the most important influencing factor.^12^, ^13^ This is consistent with our data, which indicates that FN function gradually deteriorates with increasing tumor size. The study by Falcioni et al. demonstrated a significant correlation between postoperative FN function and tumor size, which reached statistical significance (*p* < 0.0001).^12^ When the lesions are larger, unfavorable outcomes are more common. In cases where the tumor is larger than 4 cm, the proportion of unfavorable outcomes reaches 21.8%, and this relationship is statistically significant (*p* < 0.0001). Hobson et al. found that patients with better early FN function had smaller tumor diameters (average 31.6 mm) compared to patients with poorer FN function (average 35.5 mm) (*p* < 0.001). In the univariate logistic regression analysis, an increase in tumor size correspondingly increased the odds of poorer early FN function (OR = 1.09 [1.03–1.15], *p* = 0.003). Similar results were obtained in the multivariable logistic regression analysis after adjusting for age and other influencing factors (OR = 1.10 [1.03–1.17, *p* = 0.002]).^14^ The FN is closely related to tumors, and as tumors grow, the compression and stretching of the nerve intensify, increasing the possibility of intraoperative traction injuries.^15^ Furthermore, tumor compression may affect the blood supply to the nerve and can result in postoperative deterioration of nerve function in tumors. This may explain the high incidence rate of postoperative facial paralysis in patients with larger tumors.

FFC sign refers to the presence of cerebrospinal fluid between the bottom of the IAC and the outermost end of the tumor. The existence of FFC is an important radiological predictor for FN function after VS resection.^5^ Previous studies have reported that the proportion of patients with FFC sign ranged from 40% to 71%.^7^, ^16^, ^17^ In this study, 43% of the patients had FFC, which is consistent with previous reports.^7^, ^16^, ^17^ We found that among the 79 patients with FFC, 72 patients (92.4%) had favorable FN outcomes, while among the 106 patients without FFC, 72 patients (67.9%) also had favorable FN outcomes. This indicates that patients with FFC have better FN outcomes after VS resection (univariate *p* = 0.007, multivariate *p* = 0.014). Similarly, Fujita et al.^5^ observed the same trend. In the FFC group, 80.4% patients (82/102) had favorable FN outcomes after surgery, whereas in the non‐FFC group, only 63.4% patients (26/41) had favorable FN outcomes (*p* = 0.052). The preservation rate of favorable FN outcomes in the FFC group was consistently higher than that in the non‐FFC group. The difference was significant at 12 months postoperatively (96.1% vs. 82.9%, *p* = 0.013) and at 24 months (97.1% vs. 82.9%, *p* = 0.006). Rompaey et al. reported that in the absence of FFC, 29.7% of patients achieved HB grade 3 or higher in the short term, compared to only 13.0% in patients with FFC.^18^ The absence of FFC had a significant negative impact on short‐term FN function after VS resection (*p* = 0.006).

We successfully demonstrated that FFC sign is an independent factor that influences postoperative FN function in patients with VSs. The presence of a FFC confers an advantage during microsurgery because it eliminates the need for drilling the entire IAC. The distance between the tumor and fundus facilitates gaining control of the most lateral part of the tumor in the IAC and allows for easier dissection of the FN at the inferior aspect of the tumor. On the other hand, the presence or absence of FFC may be related to differences in tumor origin in VS. It is generally believed that VS can originate anywhere between the transition zone of neural Glial–Schwann cells to the nerve endings within the vestibular receptor.^19^ This variability in origin may affect the extent of tumor filling in the IAC and the degree of adhesion to the nerve. When the origin of VS is closer to the bottom of the IAC, there is a higher risk of compression on the cochlea nerve and FN, which increases the likelihood of injury.

The CSFC sign refers to the gap of cerebrospinal fluid between the tumor and the interface of the brainstem and cerebellum.^20^ This study, consistent with previous reports, discovered that the CSFC sign has a significant protective effect on postoperative FN function in patients with VS (multivariate OR = 0.07 [0.01, 0.26], *p* < 0.001). There have been few studies on the relationship between the CSFC sign and FN outcomes. Jung et al.'s^20^ study demonstrated that the CSFC sign can predict tumor adhesion and FN outcomes. They observed the presence of the CSFC sign in nine patients (34.6%). The study found that the presence of the CSFC sign (*p* < 0.002) and a well‐defined tumor capsule (*p* < 0.004) were associated with lower adhesion. Additionally, the degree of adhesion was significantly associated with long‐term FN outcomes (*p* = 0.029). Sun et al.'s study further confirmed that the disappearance of the CSFC sign is an independent factor that affects short‐term FN prognosis (multivariate OR = 0.375 [0.169–0.832], *p* = 0.016).^3^

Previous studies and clinical experience have indicated that the degree of adhesion between the FN and the tumor capsule is an independent factor that influences FN injury after VS surgery.^21^ The anatomical structure of the intracranial portion of the FN is similar to the nerve roots of the spinal cord, as it lacks a nerve sheath but is covered by the pia mater and arachnoid mater.^22^ The absence of a CSFC sign may indicate the absence of the subarachnoid space, resulting in adhesion between the tumor and the brain, brainstem, or adjacent cranial nerves.^23^, ^24^, ^25^, ^26^ Therefore, using the CSFC sign to assess the degree of tumor adhesion and predict FN outcomes is a reliable imaging prediction method.

Our study further identified a correlation between the expansion of the IAC and postoperative FN function. The larger the expansion of the IAC, the higher the probability of postoperative FN injury (multivariate OR = 8.73 [1.23, 69.48], *p* = 0.033). This finding has been confirmed in previous studies as well, with Rizk et al. demonstrating a significant correlation between postoperative facial function and enlargement of the IAC (*p* = 0.023). This correlation may be attributed to the compression of the FN by the expanding tumor at the edge of the IAC, making it challenging to identify the anatomical plane between the tumor and the FN at the IAC edge. Surgeries for softer and more easily suctionable tumors generally involve less manipulation of the FN compared to surgeries for firmer and non‐suctionable tumors. Such manipulation of the tumor typically results in traction on the FN, which can affect the postoperative outcomes.^27^

It is important to note that all the data in this study were collected from our institution, which may have been influenced by our center's experience. This model currently lacks multicenter and external validation. The sample size is relatively small and may be susceptible to bias. Additionally, this study only predicts the short‐term postoperative FN outcome, and the possibility of delayed facial paralysis still needs to be addressed.

## 5 CONCLUSION

The preoperative radiographic features, such as FFC and CSFC sign, tumor size, and the expansion of the IAC, serve as excellent predictors of postoperative FN outcomes in patients with VSs. Based on these factors, the nomogram demonstrates good predictive performance. We have also generated a web‐based calculator to facilitate clinical application: https://xudong301.shinyapps.io/dynnomapp/.

## AUTHOR CONTRIBUTIONS

Xudong Shi, Yuyang Liu, and Zehan Zhang were responsible for conducting the formal analysis, contributing to the methodology, overseeing the project, and writing the original draft of the manuscript. Xudong Shi, Bingyan Tao, Ding Zhang, and Qingyu Jiang were involved in curating the data and collecting the necessary resources. Guilin Chen, Hengchao Ma, Jiaxin Xie and Xuan Zheng were involved in the conception of the study, as well as writing and reviewing the manuscript. Yaping Feng and Jun Zhang played a supervisory role in the study, and contributed to writing, reviewing, and editing the manuscript. The article was a collaborative effort by all authors, who approved the final version of the submitted manuscript.

## CONFLICT OF INTEREST STATEMENT

The authors have no competing interests to declare that are relevant to the content of this article.

## Data Availability

The dataset and material are available from the corresponding author on reasonable request.
